# The Story of the Insane from Year to Year

**Published:** 1906-03-24

**Authors:** 


### THE STORY OF THE INSANE FROM YEAR TO YEAR.

Eighteenth Annual Series.

### THE LANARK DISTRICT ASYLUM.

At the close of the year this Asylum had 889 patients in residence. There were 128 first admissions; 69 readmissions; 12 unknown whether first or not; and three were voluntary admissions; 103 were discharged recovered; 36 discharged relieved; and 76 died. The average number resident during the year was 897, and the total number under treatment was 1,108. The recoveries stand at the very creditable percentage of 49.2 on the admissions, and the deaths at 8.4 on the average number resident. Of the deaths 16 were from cerebral and spinal diseases; 10 from bronchitis; three from pneumonia; and 12 from consumption. Of the moral causes of insanity in the year's admissions we notice that religion accounts for 10 and worry for 10. Among the physical causes intemperance in drink comes first with 33 cases, hereditary influence follows with 22, and senility with 13, while in 25 no cause could be ascertained. Dr. Laing has continued his lectures to the attendants and nurses; and seven of these have obtained the nursing certificate after a two years' course, which in this Asylum carries with it a bonus of £2; but we do not learn that Dr. Kerr has instituted a complete three years' course of training. We are glad to find that the Visiting Committee have decided to erect a sanatorium for the treatment of 26 consumptive patients. In fact the building is probably finished by this time, and it is not without reason that the Board "hope it will prove of great advantage in enabling them not only to ameliorate the condition of patients suffering from this disease, but also to check its spread." The weekly charge to the Unions is 8s. 9d., and private patients pay at the rate of £32 a year. A similar charge is made for paupers admitted from outside the district boundaries. Dr. Kerr has three assistant medical officers, and the Board "record their sense of Dr. Kerr's ability and conscientiousness in the management of the Asylum, and of the loyal way in which he is seconded by those who assist him."

